# Does interacting with trustworthy people enhance mindfulness? An experience sampling study of mindfulness in everyday situations

**DOI:** 10.1371/journal.pone.0215810

**Published:** 2019-04-26

**Authors:** Ravi S. Kudesia, Christopher S. Reina

**Affiliations:** 1 Department of Human Resource Management, Fox School of Business, Temple University, Philadelphia, PA, United States of America; 2 Department of Management, School of Business, Virginia Commonwealth University, Richmond, VA, United States of America; Middlesex University, UNITED KINGDOM

## Abstract

Mindfulness is known to increase after meditation interventions. But might features of our everyday situations outside of meditation not also influence our mindfulness from moment-to-moment? Drawing from psychological research on interpersonal trust, we suggest that interacting with trustworthy people could influence the expression of mindfulness. And, extending this research on trust, we further suggest that the influence of trustworthy social interactions on mindfulness could proceed through two pathways: a particularized pathway (where specific interactions that are especially high (or low) in trustworthiness have an immediate influence on mindfulness) or a generalized pathway (where the typical level of trustworthiness a person perceives across all their interactions exerts a more stable influence on their mindfulness). To explore these two pathways, study participants (*N* = 201) repeatedly reported their current levels of mindfulness and their prior interactions with trustworthy leaders and teammates during their everyday situations using an experience sampling protocol ($\bar{n}$ = 3,605 reports). Results from mixed-effects models provide little support for the particularized pathway: specific interactions with trustworthy leaders and teammates had little immediate association with mindfulness. The generalized pathway, however, was strongly associated with mindfulness—and remained incrementally predictive beyond relevant individual differences and features of situations. In sum, people who typically interact with more trustworthy partners may become more mindful.

## Introduction

Mindfulness has emerged as a mental state associated with positive psychological outcomes, including reduced stress and improved cognitive functioning [1–3]. As such, scholars have good cause to consider its antecedents. What can be done to help people increase their mindfulness levels, and thereby better obtain these positive outcomes? To date, the primary answer has been mindfulness meditation training. As mindfulness itself garnered scholarly attention in large part due to such meditation trainings [4], it is unsurprising that research has predominantly emphasized this antecedent. Supportive findings indeed show that meditation practitioners report higher mindfulness levels than do non-practitioners [5]. Yet, outside of meditation trainings, the domain of research on antecedents of mindfulness remains “remarkably thin” [6].

One promising set of antecedents concerns social interactions. For instance, in clinical psychology contexts, scholars emphasize how although mindfulness training appears to operate by improving attentional functioning, its benefits may stem largely from patient-therapist interactions [7]. Similarly, in traditional contexts, mindfulness is seen as best maintained within a community of practice, known as a sangha, where the social climate is itself conducive to mindfulness [8]. Although limited, promising empirical work has found that positive and supportive relationships with parents [9] and workplace supervisors [10] may underlie and help account for between-person differences in mindfulness levels.

In the present study, we hope to contribute to this small, but growing literature on social interactions as an antecedent of mindfulness. We do so by drawing from psychological research on trust. Trust is defined as the intention to accept vulnerability based on positive expectations about the intentions or behavior of another person [11]. When people trust someone, they are willing to let down their guard because they feel that this trusted other will not take advantage of them. Trust is what allows a student to delegate work on a class project, based on her belief that all teammates will complete their respective parts—it is what allows an employee to confide in his leader, feeling assured that what he says will remain private.

There are many reasons why one person might trust another, such as their tolerance for risk or sensitivity to betrayal [12]. But how much we trust a person also depends in large part on how trustworthy we think that person is [13]. Our perceptions of a person’s trustworthiness are based on three characteristics: ability (is the person skilled enough to perform needed actions?), benevolence (does the person care enough to act in my best interest?), and integrity (does the person act in accordance with sound principles?) (see [14]). We are willing to let down our guard around a person to the extent that we perceive them as trustworthy, that is, having ability, benevolence, and competence.

In what follows, we extend research on trust into the realm of mindfulness by elaborating two pathways through which interactions with trustworthy partners could influence mindfulness. We then empirically explore this idea, that when people interact with others whom they deem to be trustworthy, they are thus empowered to become more mindful—and when they interact with others they deem to be less than trustworthy, they more readily experience reduced mindfulness.

### Components of mindfulness

Mindfulness, like trust, entails a kind of vulnerability, whereby people are willing to remain accepting and open to their internal and external experiences—including their negative thoughts and feelings [15]. Mindfulness entails a way of embracing vulnerability to experiences that integrates metacognition and attention [16]. When people are mindful, they do not ruminate on and get lost in their present-moment experiences. Instead, they engage a metacognitive mode of processing, whereby they mentally “step back” and notice their thoughts and feelings about experiences from a distant perspective [17,18]. This helps them detach from unhelpful appraisals of experiences, recognize that their thoughts and feelings do not constitute the final truth of any experience, and consider the value of other ways of interpreting experiences [16]. As a result of this metacognitive process, they can allocate fuller and more thorough attention to their full range of ongoing experiences, rather than selecting out and avoiding particular aspects of it [19,20].

Taken in sum, *mindful attention* toward experiences (i.e., allocating a fuller and more thorough attention to them) and *mindful metacognition* toward the thoughts and feelings elicited by these experiences (i.e., stepping back and noticing them from a distant perspective) capture the two key components of mindfulness as it relates to positive outcomes across several key psychological domains [16,21,22]. Although some scholars have included various additional components (cf. [23]), these two components of attention and metacognition adequately and parsimoniously capture the state of mindfulness—and have the additional benefit of aligning closely with a common operational definition of mindfulness [22] as well as traditional accounts [24].

### Mindfulness and trust

It is possible that mindfulness—as a way of engaging with present-moment experiences and relating to the resulting thoughts and feelings—may be shaped by what those experiences entail.

To be sure, people can be mindful in any type of situation, whether it is pleasant or unpleasant, whether it is individual or social in nature. But it is not equally *easy* to be mindful in all types of situations, as personal experience can readily attest. Certain situations and certain people are more likely to trigger mindless responses, like anxiety or boredom, that draw our attention away from experiences and prompt us to get wrapped up in our thoughts and feelings. This is one of the reasons why in clinical contexts, mindfulness is first trained on neutral stimuli before being turned toward the more negative stimuli that underlie anxiety disorders: it is easier to be mindful in the face of supportive stimuli (e.g., [25]). It stands to reason that the same logic may apply in the context of everyday situations, where the stimuli often come from social interactions.

When social interactions are with trustworthy partners, people may thus become more likely to be mindful. To understand why, we must consider the function of trust in social interactions. Trust exerts a direct and sizeable influence on the way we engage with experiences in terms of how we bring our attention to them and relate to the thoughts and feelings they elicit [12,26]. Most importantly, trust entails expectations about how a choice to be vulnerable to someone in the present moment will exert consequences in the future [11]. It therefore requires that we shift our attention from the present to the future and concern ourselves with the set of negative hypothetical events that could occur if our trust were to be violated. It particularly involves judgments about how sensitive we will be to betrayal and potential loss, often drawing from first-hand prior experience about the strong negative emotions that follow from trust violations [12,27].

Accordingly, people who cannot trust their leaders at work report being less able to focus their attention—and end up scaling back the scope of work they perform as a result, including work that would have helped their colleagues [28]. In contrast, when people experience trust, they do not need to constantly predict how each and every action of untrustworthy others could hypothetically lead to negative future outcomes—and whether they are willing to bear the risk of such betrayal and loss. They can instead remain more firmly grounded in their current experiences and interact more freely with others in the present moment, sharing and receiving richer information that helps them broaden the scope of their attention and consider alternate interpretations of experiences [28–30].

As such, interactions with trustworthy partners may increase mindfulness: helping people attend fully to present-moment experiences and relate to their thoughts and feelings from a more distant perspective. To be sure, mindfulness may be more *beneficial* in situations in which trust is lacking than where trust is plentiful, because situations in which trust is lacking can contain the starkest risks. But this does not mean that mindfulness is therefore automatically more *likely* in these situations. The present study is not focused on the question of when mindfulness will produce more beneficial consequences, but on its antecedents: when mindfulness will be most likely to arise. We suggest that because situations in which trust is lacking contain negative stimuli that are more difficult to engage with, people will likely experience less mindfulness in them relative to situations where trust is high. Because perceiving one’s interaction partners as trustworthy is the key stimulus that makes trust possible in a situation, it therefore becomes important to clarify the pathways through which perceptions of trustworthiness in interactions could influence mindfulness.

### Generalized and particularized pathways of trustworthiness

Much of the relevant psychological literature has taken a “levels and referents” approach to this question [26]. In this approach, trust can be studied at various levels: a person can individually trust another person, but it is equally possible for a sports team to collectively put their trust in their star player. Conversely, trust can also apply to various referents: an employee might trust her teammate as an individual, but she could also trust her organization to do the right thing as a collective. A good deal of important work has explored the factors involved with these various levels and referents, where the trustor and trustee can be either individual or collective [12,26].

A complementary body of sociologically-oriented work, however, suggests that trust can exist without any specific referent (e.g., [31–33]). Whereas a person’s trust in a specific referent is called particularized trust, because the trust is particular to a specific interaction partner, this work also discusses generalized trust, which concerns how much a person typically trusts most people [34]. This generalized form of trust is important because it signifies a level of social capital at which people in a collective can organize themselves most productively [34–36].

Building on this distinction, we suggest that there are two pathways by which trustworthy social interactions could influence mindfulness: a *particularized pathway* driven by specific interactions and a *generalized pathway* driven by typical patterns of interactions. In the particularized pathway, a single interaction with a person that is especially trustworthy could immediately enhance mindfulness, akin to a priming effect. For instance, relative to the typical everyday social interaction, when we interact with someone we find truly trustworthy, we may be able to have deeper energizing conversations that effortlessly draw us into the interaction and make us more aware of our emotional experiences from moment to moment (e.g., [37]). In contrast, the generalized pathway is not about any particular interaction, but is about the typical pattern spanning across all of a person’s interactions. For instance, a person who typically sees his interaction partners as trustworthy may feel more at home and experience a greater sense of belongingness and security that lets him more fully attend to his experiences [31].

The difference between the particularized and generalized pathways is akin to the difference between receiving a bonus and having a high salary—both entail an increase in wealth that will influence a person’s wellbeing. But they exert different types of influence. We thus expect the influence of trustworthiness on mindfulness to be positive across both pathways, an expectation called “local homogeneity” [38]. But it is not necessary that a relation explored across these two pathways must be homogeneous: in some cases, only one pathway could be significant or the two pathways could even be significant in opposite directions [39,40]. For example, completing a single workday that had particularly high time pressure might enhance wellbeing by making a person feel accomplished, but working in a job with a generally high level of time pressure might erode wellbeing by causing burnout. In this case, we expect that mindfulness will be positively associated with both the particularized and generalized pathways of trustworthy interactions.

### The present study

The present exploratory study is intended to contribute to the growing literature on the social antecedents of mindfulness, specifically by way of research on interpersonal trust. In line with recent methodological recommendations, we do so using an experience sampling design [39–42]. In this experience sampling design, participants repeatedly report their current levels of mindfulness and the trustworthiness of their recent interactions, spanning over a period of time that includes many situations. This design allows one to statistically distinguish between the particularized pathway of trust (where specific interactions with partners who are especially trustworthy or not for a participant immediately influence mindfulness) and the generalized pathway (where the average level of trustworthiness a participant perceives across all of his or her interactions exerts a more stable influence on mindfulness) [39–42].

In our design, participants reported the trustworthiness of their interactions with leaders as well as with teammates. Having participants report about these two distinct referents (leaders and teammates) offers two primary advantages. First, it increases the sample size because even if a participant did not interact with a leader at a particular measurement occasion, they might have interacted with a teammate, or vice versa. Including two referents thus increases the odds that participants will have a social interaction about which they can report. Second, it also affords an internal replication logic wherein the expected relation between trustworthiness and mindfulness is tested across two distinct social referents. This reduces the likelihood that any findings may be driven by factors unique to one kind of interaction partner, such as the relative status of leaders or the familiarity of teammates. We selected leaders and teammates because meta-analyses show that these two referents contribute unique influence on relevant outcomes and can be assessed by existing validated measures [43].

## Method

In this study, we explore an expected positive association between trustworthy interactions and mindfulness components, which may occur through particularized and generalized pathways, and which we examine empirically using an experience sampling design, as detailed below.

### Participants and procedure

Participants (*N* = 201) were undergraduates at a public university on the East Coast and received course credit for participating (56% of participants were female, the average age was 23, and 40% identified as White, 20% as Black, and 15% as Asian). We received approval for this study from the Institutional Review Board at Virginia Commonwealth University (HM20008881). One of the researchers explained the study in person to potential participants and responded to any questions before they either read and provided their informed consent electronically or opted to participate in an alternate assignment to obtain the course credit.

Participants reported their trait mindful attention and mindful metacognition approximately two weeks before the experience sampling began. These trait mindfulness measures were also adapted for experience sampling and will be described shortly. Participants received prompts for experience sampling three times a day (9:30 AM, 2:30 PM, 7:30 PM) over their phone for eight consecutive weekdays. This schedule was designed to obtain samples during academic and non-academic times, as study participants frequently work outside of class hours: 52% had at least one year of full-time work experience and 19% had more than five years.

After opening the survey, participants were asked to rate their mindfulness levels immediately prior to receiving the survey notification. They were then asked to think back over the time window since the last survey—or since the start of the day for the morning survey—and respond to items using only experiences within that window. They were asked separately whether they interacted with someone who they considered a leader or teammate. If they replied in the affirmative, they were asked to specify the respective interaction and to answer the trustworthiness items with that interaction in mind. Common interactions with leaders entailed managers, executives, and recruiters in the workplace, coaches of sports teams, and professors, academic advisors, and residence hall advisors in the university. Common interactions with teammates entailed fellow members of workplace teams, volunteer organizations, sports teams, and class project teams.

Participants finally reported their overall satisfaction with their total social interactions over the entire time window, which, as will be discussed below, constituted a control variable. Using conversational norms of narrative sequence and specific referents (i.e., how do you feel now, what did you do before, who with, how satisfied are you about it all) helped to reduce order issues whereby reporting trust could retrospectively bias reports of mindfulness [44].

### Response rate

Participants produced 3,605 total experience sampling responses (a 75% response rate), with an average participant response of approximately 18 and a median response of 20. In these responses, participants interacted with a leader 33% of the time ($\bar{n}$ = 1,183) and with a teammate 31% of the time ($\bar{n}$ = 1,132). To ensure that participants did not underreport the frequency of these interactions to shorten the survey length (as questions about trust with the leaders and teammates were necessarily skipped if participants had no such interactions), we included a display logic that added extra questions to the survey in place of any skipped trust questions. Based on established sample size benchmarks, this response rate adequately powered our study to detect the expected fixed effects of trustworthiness on mindfulness [45].

### Measures

All measures items were assessed on a 7-pt Likert-style scale and appear in S1 File.

#### Mindfulness

Participants reported their levels of both mindfulness components in response to the prompt, “Please rate your state of mind *immediately before* receiving the notification for this survey.” They reported their *mindful attention* using the five-item state Mindful Attention and Awareness Scale (e.g., “I was finding it difficult to stay focused on what is happening (reverse-coded);” [19]). They reported their *mindful metacognition* using five items adapted from the Experiences Questionnaire (e.g., “I was able to separate myself from my thoughts and feelings;” [46]). The former is the most widely-utilized measure of mindful attention and the latter is among the few measures of mindful metacognition that is face-valid and validated for use on student populations in non-clinical settings [17].

#### Trustworthiness

Participants were then asked to think back over the time window prior to the current survey (or since the start of the day for the morning survey) and respond to subsequent items using only experiences within that time window. They were separately asked whether they interacted with a leader and a teammate. If they replied affirmatively, participants identified the specific leader and/or teammate via free response and reported their trustworthiness in response to the prompt, “To what degree do the following statements describe how you felt during this interaction with the leader/teammate?” They reported *leader trustworthiness* using a six-item scale (e.g., “My needs were important to the leader;” [47]). And they reported *teammate trustworthiness* using a five-item scale (e.g., “We counted on each other to fully live up to our word;” [48]). Both scales are widely used and well-validated with respect to content and criterion validity in naturalistic contexts, including with student samples like ours (e.g., [49,50]).

#### Satisfaction with interactions

Any observed relation between the trustworthiness of a social interaction and mindfulness could be confounded by other features of the interaction or by other interactions altogether. To address this issue, participants reported their overall satisfaction with all of their social interactions in the time window. Satisfaction is a global evaluation that includes cognitive appraisals and affective experiences in a time window (see [51]). Participants responded to the prompt, “Thinking about all of your experiences since the last survey, please rate your agreement with the following statement: I feel satisfied with my social interactions,” to evaluate all their interactions in the time window [52]. Such single-item measures of satisfaction are often used, as the concept of satisfaction is readily accessible to and easily understood by participants. Such measures also demonstrate strong criterion validity relative to multi-item measures [53,54].

### Analytical approach

Because our dataset contained multiple experience sampling reports collected over time and nested within a single participant, we analyzed our data using mixed-effects modeling with the linear and nonlinear mixed effects models (nlme) package for the R statistical software [55]. We modeled the particularized pathway by person-mean centering leader trustworthiness and teammate trustworthiness variables as Level 1 predictors. Person-mean centering makes these variables reflect the effect of specific interactions that are particularly trustworthy or not for a participant [56]. We retained the average values of leader trustworthiness and team trustworthiness for each participant as Level 2 predictors to represent the generalized pathway, which reflect the effect of typical levels of trustworthiness, and were grand-mean centered to improve interpretability.

We tested whether accounting for time improved model fit by sequentially estimating linear, quadratic, and cubic fixed effects of time on mindfulness, adding a random slope for the effect of time across participants, and then modeling first-order autocorrelated errors, finding justification to include a fixed effect and random slope for the linear time variable [57]. This procedure helps detrend our data, removing any artifacts such as whether participants started to report greater levels of trust over the course of the study.

We also added random slopes for the person-mean centered trustworthiness predictors, as doing so reduces the likelihood of false positives [58] and included a random slope as well for satisfaction with interactions, as its inclusion improved fit in at least some of the models. To examine whether trustworthy interactions predict state mindfulness levels incrementally beyond one’s general tendency to be mindful, we added trait mindfulness (which was self-reported prior to the experience sampling period) as a Level 2 predictor. And given mixed evidence that gender may influence trust dynamics [59–62], we also included participant gender as an additional Level 2 predictor. None of the model results change with the inclusion or exclusion of this variable.

We present our results through three nested models. First, with only the particularized pathway. Second, with both the particularized and generalized pathway. Third, with the particularized and generalized pathway, along with the satisfaction with interactions, trait mindfulness, and gender variables. This third model helped determine the incremental predictive validity of trustworthiness on state mindfulness beyond a relevant feature of the situation and participant individual differences. The system of equations for this third stage model appears below. For all models, we report R^2^_GLMM_ in the tables to describe the total variance explained by the fixed effects alone (marginal R^2^_GLMM_) and by the fixed and random effects combined (conditional R^2^_GLMM_) and display the variance components to provide information about the variance explained at each level [63].

Level 1:

$${Mindfulness}_{ij}=\beta_{0j}+\beta_{1j}{\left(Time\right)}_{ij}+\beta_{2j}({Particularized\;Trustworthiness)}_{ij}+\beta_{3j}({Satisfaction\;with\;Interactions)}_{ij}+r_{ij}$$

Level 2:

$$\beta_{0j}=\gamma_{00}+\gamma_{01}{\left(Generalized\;Trustworthiness\right)}_{j}+{\gamma_{02}(Trait\;Mindfulness)}_{j}+{\gamma_{03}(Gender)}_{j}+u_{0j}$$

$$\beta_{1j}=\gamma_{10}+u_{1j}$$

$$\beta_{2j}=\gamma_{20}+u_{2j}$$

$$\beta_{3j}=\gamma_{30}+u_{3j}$$

## Results

Descriptive statistics and correlations among study variables appear in Table 1. All measures demonstrated adequate multilevel reliability at the between-person (R_KRN_ = .94–.95) and within-person (R_CN_ = .84–.91) levels [64,65]. Intraclass correlations show that 39–45% of the variance in the experience sampling variables (mindful attention, mindful metacognition, leader trustworthiness, teammate trustworthiness, satisfaction with interactions) occurs between-person, whereas 55–61% occurs within-person (reflecting situational variability and measurement error).

**Table 1:** Descriptive statistics and correlations among study variables.

	Mean	SD	ICC(1)	1	2	3	4	5	6	7	8
1. Trait Mindful Attention	4.22	1.03	—-	(.84)							
2. Trait Mindful Metacognition	4.97	0.91	—-	.36***	(.82)						
3. Gender (0 = Male, 1 = Female)	0.57	0.49	—-	–.08	–.24***	—-					
4. Mindful Attention	4.61	1.53	.45	.38***	.29***	–.03	(.95/.84)	.31***	.03	–.01	.13***
5. Mindful Metacognition	4.82	1.36	.44	.32***	.39***	–.03	.52***	(.95/.84)	.04	.04	.17***
6. Leader Trustworthiness	5.74	1.23	.43	–.02	.00	.19*	.11	.24**	(.94/.91)	.15***	.12***
7. Teammate Trustworthiness	5.86	1.07	.48	.00	.04	.15	.17*	.19*	.54***	(.95/.86)	.17***
8. Satisfaction with Interactions	5.36	1.40	.39	.25***	.24***	.12	.35***	.48***	.25***	.33***	—-

*Note*: *N* = 201 participants, $\bar{n}$ = 3,605 responses for variables (3,4,7), 1,183 for variable 5, and 1,132 for variable 6. Variables listed above the horizontal line (1–3) represent initial assessment measures, whereas variables listed below the horizontal line (4–8) represent experience sampling measures. For experience sampling measures, correlations below the diagonal were conducted on average values (between-person results) while correlations above the diagonal were conducted on person-mean centered values (within-person results). Reliabilities for scales appear along the diagonal, displayed as (α) for trait-level reliability above the horizonal line and as (R_KRN_/R_CN_) for multilevel reliability below the horizontal line.

* *p* < .05

** *p* < .01

*** *p* < .001

### Mindfulness and leader trustworthiness

As shown in Table 2, the particularized pathway of leader trustworthiness did not significantly predict mindful attention, γ = .03 [95% CI:–.07, .13], *t*(999) = 0.53, *p* = .600, while the generalized pathway was marginally significant: γ = .16 [95% CI:–.02, .34], *t*(177) = 1.78, *p* = .078. The particularized pathway of leader trustworthiness similarly did not significantly predict mindful metacognition, γ = .01 [95% CI:–.08, .11], *t*(999) = 0.31, *p* = .757, while the generalized pathway was significant: γ = .25 [95% CI: .08, .41], *t*(177) = 2.96, *p* = .004. Thus, we find no evidence for the particularized pathway of leader trustworthiness and find evidence for the generalized pathway only for the metacognition component.

**Table 2:** Mixed-effects modeling for leader trustworthiness.

	Model 1–1	Model 1–2	Model 1–3	Model 2–1	Model 2–2	Model 2–3
Intercept	4.69***	4.69***	4.70***	4.76***	4.76***	4.76***
	[4.50,4.88]	[4.50,4.88]	[4.44,4.96]	[4.57,4.94]	[4.59,4.95]	[4.52,5.01]
Time	–0.01	–0.01	–0.03	0.03*	0.03*	0.02
	[–0.05,0.02]	[–0.05,0.02]	[–0.06,0.01]	[0.00,0.06]	[0.00,0.06]	[–0.01,0.05]
Particularized Leader Trustworthiness	0.05	0.05	0.03	0.05	0.04	0.01
	[–0.05,0.15]	[–0.05,0.15]	[–0.07,0.13]	[–0.04,0.14]	[–0.05,0.13]	[–0.08,0.11]
Generalized Leader Trustworthiness		0.16+	0.16+		0.26**	0.25**
		[–0.02,0.35]	[–0.02,0.34]		[0.09,0.44]	[0.08,0.41]
Gender			0.00			–0.02
			[–0.33,0.32]			[–0.33,0.29]
Trait Mindful Attention			0.34***			
			[0.18,0.50]			
Trait Mindful Metacognition						0.43***
						[0.28,0.58]
Satisfaction with Interactions			0.21***			0.19***
			[0.13,0.29]			[0.11,0.27]
Dependent Variable	Mindful Attention			Mindful Metacognition		
Random Effects						
Level 2 Variance	0.96	0.95	0.84	1.02	1.00	0.88
Time Slope	.08	.08	.07	.08	.08	.07
Leader Trustworthiness Slope	.25	.25	.27	.26	.26	.30
Satisfaction Slope			.18			.27
Residual Variance	1.15	1.15	1.12	0.99	0.99	0.94
Model Information						
AIC	4047.21	4049.11	4019.16	3758.70	3755.16	3690.39
Log Likelihood	–2013.60	–2013.55	–1991.58	–1869.35	–1866.58	–1827.20
Marginal R^2^_GLMM_	.00	.01	.07	.00	.03	.13
Conditional R^2^_GLMM_	.45	.45	.48	.51	.51	.56
Sample Size at Level 2	1183	1183	1183	1183	1183	1183
Sample Size at Level 1	181	181	181	181	181	181

*Note*. Coefficients are unstandardized, 95% confidence intervals for estimates appear in parentheses below estimates in lieu of standard errors.

+ *p* < .10

* *p* < .05

** *p* < .01

*** *p* < .001

### Mindfulness and teammate trustworthiness

As shown in Table 3, the particularized pathway of teammate trustworthiness did not significantly predict mindful attention, γ = –.05 [95% CI:–.15, .06], *t*(962) = –0.90, *p* = .372, while the generalized pathway was significant: γ = .23 [95% CI: .02, .43], *t*(163) = 2.19, *p* = .030. The particularized pathway of teammate trustworthiness similarly did not significantly predict mindful metacognition, γ = .03 [95% CI:–.07, .13], *t*(962) = 0.64, *p* = .522, while the generalized pathway was significant: γ = .26 [95% CI: .08, .44], *t*(163) = 2.83, *p* = .005. Thus, we find no evidence for the particularized pathway of teammate trustworthiness but find evidence for the generalized pathway for both the attention and metacognition components.

**Table 3:** Mixed-effects modeling for teammate trustworthiness.

	Model 3–1	Model 3–2	Model 3–3	Model 4–1	Model 4–2	Model 4–3
Intercept	4.77***	4.77***	4.70***	4.76***	4.76***	4.73***
	[4.57,4.98]	[4.56,4.97]	[4.44,4.97]	[4.56,4.96]	[4.56,4.95]	[4.49,4.97]
Time	0.00	0.00	0.00	0.05**	0.05**	0.04**
	[–0.03,0.04]	[–0.03,0.04]	[–0.04,0.03]	[0.02,0.08]	[0.02,0.08]	[0.01,0.08]
Particularized Teammate Trustworthiness	0.00	0.00	–0.05	0.07	0.07	0.03
	[–0.10,0.11]	[–0.10,0.11]	[–0.15,0.06]	[–0.03,0.18]	[–0.03,0.17]	[–0.07,0.13]
Generalized Teammate Trustworthiness		0.26*	0.23*		0.31**	0.26**
		[0.04,0.47]	[0.02,0.43]		[0.010,0.49]	[0.08,0.44]
Gender			0.07			0.01
			[–0.25,0.40]			[–0.28,0.30]
Trait Mindful Attention			0.35***			
			[0.19,0.51]			
Trait Mindful Metacognition						0.40***
						[0.26,0.55]
Satisfaction with Interactions			0.22***			0.20***
			[0.13,0.30]			[0.10,0.29]
Dependent Variable	Mindful Attention			Mindful Metacognition		
Random Effects						
Level 2 Variance	0.99	0.99	0.88	1.02	1.00	0.80
Time Slope	.09	.09	.07	.10	.10	.08
Teammate Trustworthiness Slope	.20	.20	.16	.24	.24	.23
Satisfaction Slope			.18			.32
Residual Variance	1.10	1.10	1.08	0.97	0.97	0.92
Model Information						
AIC	3766.58	3765.84	3743.50	3517.44	3512.61	3453.49
Log Likelihood	–1873.29	–1871.92	–1853.75	–1748.72	–1745.30	–1708.75
Marginal R^2^_GLMM_	.00	.02	.08	.01	.04	.13
Conditional R^2^_GLMM_	.45	.45	.47	.48	.48	.54
Sample Size at Level 2	1132	1132	1132	1132	1132	1132
Sample Size at Level 1	167	167	167	167	167	167

*Note*. Coefficients are unstandardized, 95% confidence intervals for estimates appear in parentheses below estimates in lieu of standard errors.

* *p* < .05

** *p* < .01

*** *p* < .001

### Adjustment for multiple comparisons

Testing the two pathways (particularized, generalized) across two referents (leader, teammate) to predict two mindfulness components (attention, metacognition) is beneficial from an internal replication standpoint, but also raises multiple comparison concerns. Namely, our expectation that trust enhances mindfulness was explored using eight different statistical tests, which increases the chance that any of these tests could have reached significance purely by chance. In addition to the other steps taken to reduce false positives, we thus adjusted *p*-values from these eight tests for a false discovery rate of α = .05 using the procedure introduced by Benjamini and Hochberg [66]. After doing so, none of the particularized pathways were significant, the generalized pathway of teammate trustworthiness on mindful attention became marginally significant (*p* = .079), the previously marginal generalized pathway of leader trustworthiness on mindful attention became non-significant (*p* = .154), and the generalized pathway of leader trustworthiness and teammate trustworthiness both remained significant for mindful metacognition (*p* = .021). This lends additional credibility to the association between the generalized pathway of trustworthiness and mindful metacognition.

## Discussion

As research documenting the benefits of mindfulness continues to accrue, research that more fully specifies its antecedents must keep pace as well: the better we understand how to enhance mindfulness in everyday situations, the more practically valuable research on its outcomes becomes. Although previous work has explored positive relationships as antecedents of between-person differences in mindfulness (e.g., [9,10]), to our knowledge, no published study has empirically examined whether social interactions function as antecedents of mindfulness at the within-person level. In the present study, we explore this possibility by focusing on the role of trustworthiness and considering two pathways through which trustworthiness may influence mindfulness. We found that whereas trustworthy interactions were not associated with either of the mindfulness components through the particularized pathway based on specific interactions, they were through the generalized pathway based on typical interactions—and most robustly so for the metacognition component of mindfulness. Our study therefore suggests the value of studying social interactions as an antecedent of mindfulness and distinguishing particularized and generalized pathways when doing so.

### Contributions and limitations

Our finding that it may be easier to be mindful when we are surrounded by people we find trustworthy lends credence to clinical [7], traditional [8], and organizational [21] theories that mindfulness can be sustained through supportive contexts. We hope this study can further serve to enrich both the mindfulness and trust literatures, as well as to set the stage for additional future research on the relation between trust and mindfulness.

#### Contributions to mindfulness research

Given how seldom the antecedents of mindfulness aside from meditation trainings are studied, especially social antecedents, our within-person study offers unique insights to the mindfulness literature. For instance, organizational psychologists have offered various views of mindfulness and how it can best be promoted. On one hand, mindfulness could be something that must be broadly cultivated and that embeds itself over time into relatively stable patterns of social interactions [21,67]. On the other, mindfulness could be something that is promoted more momentarily through targeted on-the-spot micro interventions [68]. Our findings bear on these important practical questions by suggesting that, at least in regard to the trustworthiness of social interactions, mindfulness may be better enhanced by stable patterns of social interactions, rather than more specific situational interventions. A manager should think more about how to create a generalized sense of trustworthiness among employees, rather than focusing on having specific one-off interactions with employees that particularly convey a sense of trustworthiness.

Given a relative lack of research simultaneously exploring both attention and metacognition components of mindfulness, our study further suggests that these components may not be equally easily influenced by the same antecedents. This finding contributes to ongoing debate about how to best assess mindfulness. For instance, some scholars agree that the metacognition component is theoretically distinct from the attention component, but have not found empirical evidence that the two function differentially in predicting outcomes [69]. They therefore argue for assessing mindfulness using only its attention component, as a way to counteract the use of measures that include numerous facets of uncertain validity. By exploring both components together in this study, we found that regardless of whether they differentially predict outcomes, the two components are at least differentially predicted by antecedents. After correcting for multiple comparisons, we found strong support for an association between generalized trustworthiness and mindful metacognition, but not for mindful attention. This suggests the value in assessing both mindfulness components to better cover the conceptual space of mindfulness while still maintaining relative parsimony, in line with common operational definitions [22].

#### Contributions to trust research

Because psychological research on trust so often has taken a “levels and referents” approach [26], our theoretical and methodological approach to distinguishing between particularized and generalized trustworthiness perceptions could be a valuable contribution to the literature. Our approach does less to emphasize specific referents, like a particular leader or a particular teammate. It instead calls attention to the immediate effects of specific social interactions relative to the stable effects of typical patterns of interactions, the latter of which is more aligned in some ways with sociological approaches to trust [34].

To our knowledge, prior research has not studied trustworthiness using an approach such as ours, which may offer complementary insights to the levels and referents approach. Our study suggests that the consequences of trustworthiness may not best function through momentary positive social interactions, but through longer-lasting patterns of social interactions that endure with a degree of stability. This supports the idea that generalized trust can provide a sense of “ontological security” that helps people better situate themselves in their everyday life, including in a mindful manner [31]. And because sociological research has only recently started empirically examining the micro-foundations of generalized trust [70], our findings about the relative influence of generalized and particularized pathways of trustworthiness could help clarify the role played by trustworthiness perceptions and social interactions. For instance, it may be wise to pay less heed to salient but infrequent interactions with untrustworthy people and to concern ourselves more with the institutions that ensure the trustworthiness of our typical interaction [35].

#### Limitations and future directions

It has long been noted that tradeoffs exist between experimental and correlational research, with the former better establishing causality through manipulations and the latter enhancing the external validity of studies (e.g., [71]). Our intent for this study was to explore more naturalistic settings, as this is where trust is more properly manifested and because research seldom explores antecedents of mindfulness in everyday settings (cf. [72]). But because this study is correlational, it cannot be used to make causal inferences. One could even make a case for reverse causality, where instead of trustworthy interactions influencing mindfulness levels, mindfulness may bias a person’s perceptions of trustworthiness. Although we designed our experience sampling survey to avoid such order issues (e.g., collecting current mindfulness levels first and then asking about the trustworthiness of interactions over the wider preceding time window), we cannot completely rule out this possibility with our correlational experience sampling data.

We do, however, have two reasons to suggest against this reverse causality concern. First, our study concerns perceptions of trustworthiness (am I interacting with someone who has ability, benevolence, and integrity?) rather than a person’s experience of trust (am I willing to accept vulnerability from the person I’m interacting with?). People differ in their propensity to trust others, which allows for the possibility that individual differences like mindfulness could, in theory, bias perceptions of trustworthiness [13,41]. But individual difference factors known to increase people’s propensity to trust others relate more to their willingness to bear the possibility of trust violations (e.g., greater risk tolerance, less sensitivity to betrayal) than to a bias leading them to perceive others as being more trustworthy than they actually are [12]. It seems unlikely that mindfulness in particular would somehow bias perceptions, causing people to overestimate the trustworthiness of others, when mindfulness is generally thought to promote the accuracy of perceptions (cf. [20]).

Second, we can empirically assess whether mindful participants typically perceived others as more trustworthy by examining the bivariate correlations between trait mindfulness and averaged trustworthiness. Trait mindfulness represents participants’ levels of mindfulness in general, rather than in relation to any specific social situation, and was measured prior to the experience sampling period. A correlation between trait mindfulness and averaged trustworthiness variables would suggest that mindfulness consistently influenced ratings of trustworthiness across the situations reported in the experience sampling data (e.g., [39,42]). None of these correlations were statistically significant and coefficients hovered around zero (see Table 1). As such, we find no evidence that mindfulness biases perceptions of trustworthiness either upward or downward. This helps alleviate potential reverse causality concerns: in this sample, mindful people did not seem to perceive more trustworthiness in general, select into contexts that elicit more trustworthiness, or selectively report situations with greater trustworthiness.

Nonetheless, the exploratory nature of this study provides only an initial glimpse into the complex relation between mindfulness, social interactions, and trustworthiness. We suggest that there is room for substantial future contributions in this area, particularly with complementary designs to ours. Such designs could experimentally manipulate trustworthiness and examine the consequences on mindfulness, attend more deeply to the role of mindfulness in the process of forming perceptions about social interactions, or longitudinally unpack how generalized trustworthiness is developed, considering both the individual differences emphasized in the psychology literature [13,41] and the institutional factors emphasized within sociology [34,70].

## Conclusion

This study helps expand mindfulness beyond the individual mind into its social context. It suggests that although single social interactions with people that are particularly trustworthy may not influence our mindfulness, being in a context where more of our interactions are with people we deem trustworthy can facilitate the metacognitive component of mindfulness. This finding holds regardless of whether the trusted partner was a leader or a teammate and remains incrementally predictive beyond relevant features of situations and individual differences. Thus, returning to vulnerability, a defining feature of mindfulness and trust alike, it may be easier to be vulnerable internally to our thoughts and feelings when we can be vulnerable externally to the people around us.

## Supporting information

S1 File Measures. Measures used in this study.
(DOCX)

S2 File Data. Data used in this study.
(XLSX)
