# Supplementary material for: Does interacting with trustworthy people enhance mindfulness? An experience sampling study of mindfulness in everyday situations
Source: PLoS One. 2019 Apr 26;14(4):e0215810. doi: 10.1371/journal.pone.0215810 (PMC6485709; doi:10.1371/journal.pone.0215810)
Supplement: S1 File — Measures used in this study. (DOCX) [file pone.0215810.s001.docx]

**Measures**

**Trait Measures**

**Mindful Attention [1]**

1. I could be experiencing some emotion and not be conscious of it until some time later.
2. I find it difficult to stay focused on what’s happening in the present.
3. I tend not to notice feelings of physical tension or discomfort until they really grab my attention.
4. It seems I am “running on automatic,” without much awareness of what I’m doing.
5. I rush through activities without being really attentive to them.
6. I get so focused on the goal I want to achieve that I lose touch with what I’m doing right now to get there.
7. I do jobs or tasks automatically, without being aware of what I’m doing.
8. I find myself doing things without paying attention.
9. I find myself preoccupied with the future or the past.
10. I find myself listening to someone with one ear, doing something else at the same time.

**Mindful Metacognition [2]**

1. I am able to accept myself as I am.
2. I can slow my thinking at times of stress.
3. I notice that I don’t take difficulties so personally.
4. I can separate myself from my thoughts and feelings.
5. I can observe unpleasant feelings without being drawn into them.
6. I have the sense that I am fully aware of what is going on around me and inside me.
7. I view things from a wider perspective.
8. I can see that I am not defined by my thoughts.
9. I am consciously aware of a sense of my body as a whole.

**Experience Sampling Measures**

**Mindful Attention [1]**

Please rate your state of mind immediately before receiving the notification for this survey:

1. I was finding it difficult to stay focused on what is happening.
2. I was doing something without paying attention.
3. I was preoccupied with the future or the past.
4. I was doing something automatically, without being aware of what I am doing.
5. I was rushing through something without being really attentive to it.

**Mindful Metacognition [2]**

Please rate your state of mind immediately before receiving the notification for this survey:

1. I was not easily carried away by my thoughts and feelings.
2. I was not letting difficulties affect me personally.
3. I was able to separate myself from my thoughts and feelings.
4. I could notice unpleasant feelings without being drawn into them.
5. I could slow my thinking at times of stress.

**Trust in Leader [3]**

To what degree do the following statements describe how you felt during this interaction with the leader?

1. The leader seemed very capable of performing his/her job.
2. I felt very confident about the leader’s skills.
3. I liked the leader’s values.
4. Sound principles seemed to guide the leader’s behavior.
5. The leader cared about my welfare.
6. My needs were important to the leader.

**Trust in Teammate [4]**

To what degree do the following statements describe how you felt during this interaction with the teammate?

1. We absolutely respected each other’s competence.
2. Both of us showed absolute integrity.
3. We expected the complete truth from each other.
4. We were certain that we could fully trust each other.
5. We counted on each other to fully live up to our word.

**Satisfaction with Interactions [5]**

Thinking about all of your experiences since the last survey, please rate your agreement with the following statement:

1. I feel satisfied with my social interactions.

**References**

1. Brown KW, Ryan RM. The benefits of being present: Mindfulness and its role in psychological well-being. J Pers Soc Psychol. 2003;84: 822–848. doi:10.1037/0022-3514.84.4.822

2. Fresco DM, Moore MT, van Dulmen MH, Segal ZV, Ma SH, Teasdale JD, et al. Initial psychometric properties of the Experiences Questionnaire: Validation of a self-report measure of decentering. Behav Ther. 2007;38: 234–246. doi:10.1016/j.beth.2006.08.003

3. Kim PH, Ferrin DL, Cooper CD, Dirks KT. Removing the shadow of suspicion: The effects of apology versus denial for repairing competence- versus integrity-based trust violations. J Appl Psychol. 2004;89: 104–118. doi:10.1037/0021-9010.89.1.104

4. Simons TL, Peterson RS. Task conflict and relationship conflict in top management teams: The pivotal role of intragroup trust. J Appl Psychol. 2000;85: 102–111. doi:10.1037/0021-9010.85.1.102

5. Smith PC, Kendall LM, Hulin CL. The measurement of satisfaction in work and retirement. Chicago, IL: Rand–NcNally; 1969.
